# Transperineal abdominoperineal resection for anorectal melanoma: A case report

**DOI:** 10.1016/j.ijscr.2019.07.054

**Published:** 2019-07-25

**Authors:** Hiroki Hashida, Masato Kondo, Daisuke Yamashita, Shigeo Hara, Ryosuke Mizuno, Motoko Mizumoto, Hiroyuki Kobayashi, Satoshi Kaihara

**Affiliations:** a Department of Surgery, Kobe City Medical Center General Hospital, Kobe, Japan; b Department of Diagnostic Pathology, Kobe City Medical Center General Hospital, Kobe, Japan

**Keywords:** Anorectal melanoma, Metastasis, Transperineal abdominoperineal resection, Transanal total mesorectal excision

## Abstract

**Introduction:**

Anorectal melanoma is a rare type of cancer characterized by frequent metastasis. We report our experience with a case of anorectal melanoma resected via transperineal abdominoperineal resection and total mesorectal excision.

**Presentation of case:**

A 77-year-old woman visited our hospital with the complaint of melena. Colonoscopic examination revealed a black type 1 tumor measuring 5 mm in size on the dentate line of the anal canal circumference. Tumor biopsy revealed malignant melanoma, and positron emission tomography-computed tomography showed tracer accumulation within the mass but not at other sites. We performed a two-stage abdominoperineal resection surgery with lymphadenectomy via transperineal abdominoperineal resection, and pathological diagnosis indicated submucosal tumor depth. The patient was diagnosed with T2 N0 M0 stage I malignant melanoma and was followed on an outpatient basis. A 24-month follow-up computed tomography revealed multiple liver metastases.

**Discussion:**

Anorectal melanoma is associated with a very poor prognosis because of its association with early lymph node metastasis and distant metastasis. The tumor may spread beyond the main lesion and involve the surrounding mucosa and draining lymph nodes. Transperineal abdominoperineal resection is a minimally invasive procedure that can be used to achieve curative tumor resection with lymphadenectomy.

**Conclusion:**

Transperineal abdominoperineal resection appears to be a feasible approach for the treatment of anorectal melanoma.

## Highlights

• Anorectal malignant melanoma is rare and prone to metastasis.
• An anorectal melanoma was resected via transperineal abdominoperineal resection (TpAPR).
• The patient remained disease-free until the 24-month follow-up.
• TpAPR/total mesorectal excision (TME) for anorectal melanoma appears to be a feasible approach.

## 1 Introduction

Anorectal melanoma is a rare type of malignant tumor prone to hematogenous and lymphatic metastasis. Additionally, these tumors often progress to distant metastasis by the time of diagnostic confirmation, leading to poor prognosis [1,2]. Anorectal melanoma is frequently refractory to chemotherapy and resistant to radiotherapy. Therefore, the conventional treatment for local disease control involves complete surgical tumor resection [1,3]. The optimal surgical procedure for anorectal melanoma and whether abdominoperineal resection of the anorectum or wide local excision of the tumor yields a superior outcome remain matters of controversy [4,5]. However, abdominoperineal resection is currently considered to be the standard treatment for anorectal melanoma, as this procedure may prevent lymphatic spread and secure a safe resection margin.

Recent reports have described the use of transanal total mesorectal excision (TaTME) or transperineal abdominoperineal resection (TpAPR) for rectal tumors [2,6]. This approach provides better surgical visibility than does the conventional perineal approach. Here, we report a case of anorectal melanoma treated via abdominoperineal resection with the TpAPR procedure according to the SCARE criteria [7].

## 2 Presentation of case

**Fig. 1:**
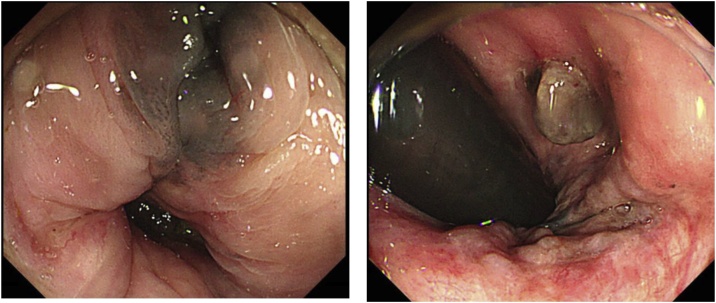
Colonoscopy findings demonstrating the manifestation of a pigmented neoplasm at the dentate line.

**Fig. 2:**
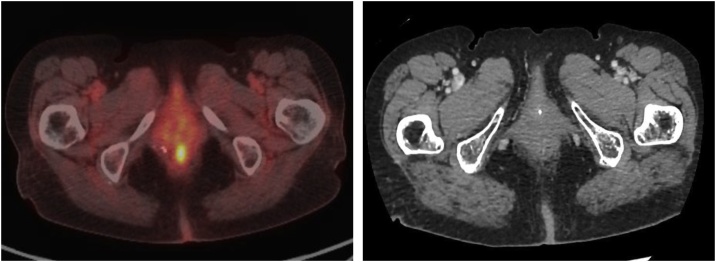
Positron emission tomography-computed tomography findings Accumulation of ^18^F-fluorodeoxyglucose is visible in a mass at the anorectal junction.

A 77-year-old woman presented at our institution with the complaint of melena. Her medical history included hypertension and cardiopathy but not melanoma. A digital rectal examination revealed a small hard mass in the anal canal. Subsequently, colonoscopic examination revealed a pigmented neoplasm measuring 5 mm that had arisen at the dentate line (Fig. 1). Tumor biopsy revealed malignant melanoma. ^18^F-Fluorodeoxyglucose (FDG) positron emission tomography (PET)-computed tomography (CT) revealed tracer accumulation in the mass at the anorectal junction but no evidence of lymph nodes or distant metastases (Fig. 2).

**Fig. 3:**
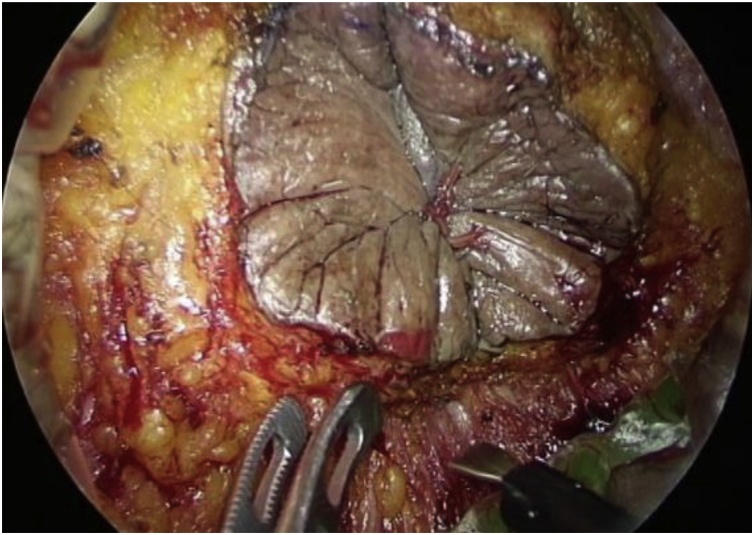
Endoscopy showing transperineal dissection in the caudal-cranial direction.

We resected the anorectal melanoma via abdomino-transperineal TME performed by two surgical teams. One team performed a laparoscopic procedure via the transabdominal approach, while the other team performed endoscopic TpAPR (equivalent to TaTME) using a multiport device (GelPoint Mini; Applied Medical, Rancho Santa Margarita, CA, USA) (Fig. 3). The laparoscopic transabdominal procedure involved dissection according to TME and high central ligation of the inferior mesenteric artery. During the endoscopic perineal procedure, a multiport device was inserted through a skin incision made around the tightly closed anus, and transperineal dissection was achieved in the caudal-cranial direction under endoscopic viewing. The adipose tissue of the ischio-anal fossa was divided to widely expose the levator ani muscle. Next, the sigmoid colon was transected with a linear stapler, and the specimen was extracted transperineally. Finally, a permanent colostomy was created laparoscopically. The total operation time was 325 min, and the total blood loss volume was negligible. The patient experienced no significant postoperative complications.

**Fig. 4:**
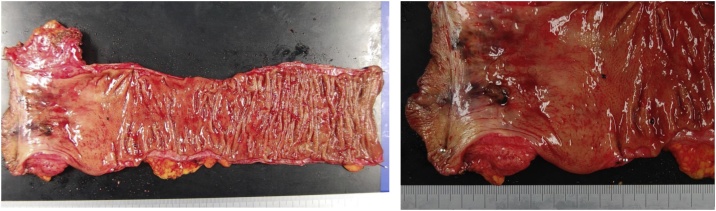
The resected specimen showing invasion of the pigmented tumor to the perirectal tissue at the anorectal junction.

**Fig. 5:**
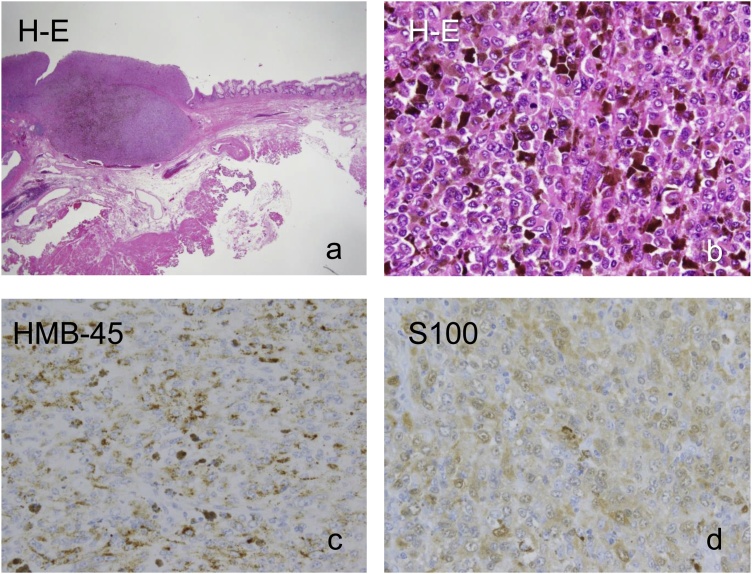
Pathological findings. a) Hematoxylin-eosin staining revealed that atypical cells formed the nests, and spread into the anal canal tissue. b) Enlarged view of tumor cells. Pigmentation was prominent, and the tumor cells had large nucleoli. c, d) Immunohistochemistry showed that tumor cells showed expression of HMB-45 and S100. Original magnification: a: 40×, b-d: 400×.

Pathologically, the tumor specimen revealed invasion to the submucosal layer. However, no tumor invasion of the resected margin (Fig. 4), and no lymph node metastases were detected. Hematoxylin-eosin staining was performed to examine tumor morphology, revealing that atypical cells formed the nests, and spread into the anal canal tissue. Pigmentation was prominent, and the tumor cells had large nucleoli (Fig. 5a, b). Other samples were immunostained for HMB-45 and S100. Immunohistochemistry showed that tumor cells showed expression of HMB-45 and S100 (Fig. 5c, d).

At a 24-month follow-up, CT revealed multiple liver metastases. Accordingly, the patient began receiving treatment with the programmed cell death protein-1 (PD-1)-specific antibody therapy nivolumab.

## 3 Discussion

Anorectal melanomas are rare and aggressive tumors with poor prognosis. The overall 5-year survival rate is 4–31%, and the reported median survival durations range from 16 to 28 months [1,3,4,8]. Although extended survival requires both early detection and diagnosis of the tumor and complete surgical resection, these factors are often delayed. Symptomatically, anorectal melanoma often presents with anal bleeding, discomfort or pain, and anal lesions.

As noted in the Introduction, no consensus has been reached regarding the preferred surgical approach for anorectal melanoma. Many researchers suggest that abdominoperineal resection is superior to local resection because it can better control lymphatic spread and achieve negative margins [9,10]. Moreover, laparoscopic abdominoperineal resection may achieve disease control while reducing morbidity [10,11]. In contrast, other researchers often recommend wide local excision as a palliative alternative to unnecessarily invasive radical surgery [12]. Although surgical resection with a safe margin is considered to be typical, the area of resection and lymph node dissection have not yet been established. In this regard, several studies suggest that abdominoperineal resection may be a useful treatment option that can control the lymphatic spread and ensure wide safety margins [4,5,8,13]. In contrast to conventional options, the TaTME technique has recently been identified as a minimally invasive surgical option that may improve the surgical and oncological outcomes of patients with rectal cancer [2,6,14]. Notably, this approach enables the achievement of safe resection margins with good visibility, particularly in cases where a large tumor and narrow pelvis present challenges to conventional resection and increase the risk of a non-curative surgical outcome. However, TaTME via a single port may be complicated by limited maneuverability and anatomical landmarks [7,15]. Accordingly, in this case, two teams performed transperineal TME in two stages: the laparoscopic transabdominal approach and endoscopic transperineal approach. This two-stage procedure allowed careful exposure of the extralevator field and sufficient resection margins to control the lymphatic spread. This procedure may also have allowed us to avoid technical complications such as injury to the pelvic nerve plexus, urethra, and/or vaginal wall. Our experience suggests that this two-step procedure is useful for avoiding the risk of anatomical complications associated with standard TaTME [8,16].

Given the rarity of anorectal melanoma, it is difficult to conduct randomized controlled trials of adjuvant chemotherapies and new treatment modalities [9,11]. To date, only one other report has described the use of transperineal TME for the treatment of anorectal melanoma [17]. We note that we did not introduce adjuvant therapy in the present case because the patient was elderly. Despite the limitations associated with rare disease, however, recent studies have yielded dramatic developments in chemotherapy for malignant melanoma. For example, antibodies targeting PD-1 and cytotoxic T-lymphocyte antigen 4, as well as an inhibitor of B-Raf, have all been introduced for the treatment of malignant melanoma [[10], [11], [12],[18], [19], [20]]. The combination of complete surgical resection and powerful chemotherapy would be expected to improve the outcomes of patients with anorectal melanoma. However, an additional accumulation of cases is needed to confirm the usefulness of this surgical technique.

## 4 Conclusion

TpAPR for anorectal melanoma appears to be a feasible approach. Additionally, new immuno-chemotherapeutic agents are expected to yield improved outcomes for patients with anorectal malignant melanoma.

## Funding

None.

## Ethical approval

This case report was approved by the Kobe City Medical Center General Hospital Review Board. (#zn190604).

## Consent

We obtained consent to publish a case reports and from the patient.

## Author contribution

HH drafted the manuscript, and MK, RM, MM, HK and SK had revised the manuscript critically. DY and SH had revised the histopathological findings. All authors contributed to study concept or design at this submission and approved the final manuscript.

## Registration of research studies

N/A.

## Guarantor

Corresponding author; Hiroki Hashida.

## Provenance and peer review

Not commissioned, externally peer-reviewed.

## Declaration of Competing Interest

None.
